# Supplementary material for: The Influence of Indisulam on Human Immune Effector Cells: Is a Combination with Immunotherapy Feasible?
Source: Pharmaceutics. 2025 Mar 14;17(3):368. doi: 10.3390/pharmaceutics17030368 (PMC11945250; doi:10.3390/pharmaceutics17030368)
Supplement: Supplementary file 1 [file pharmaceutics-17-00368-s001.zip › pharmaceutics-3161346-supplementary.pdf]

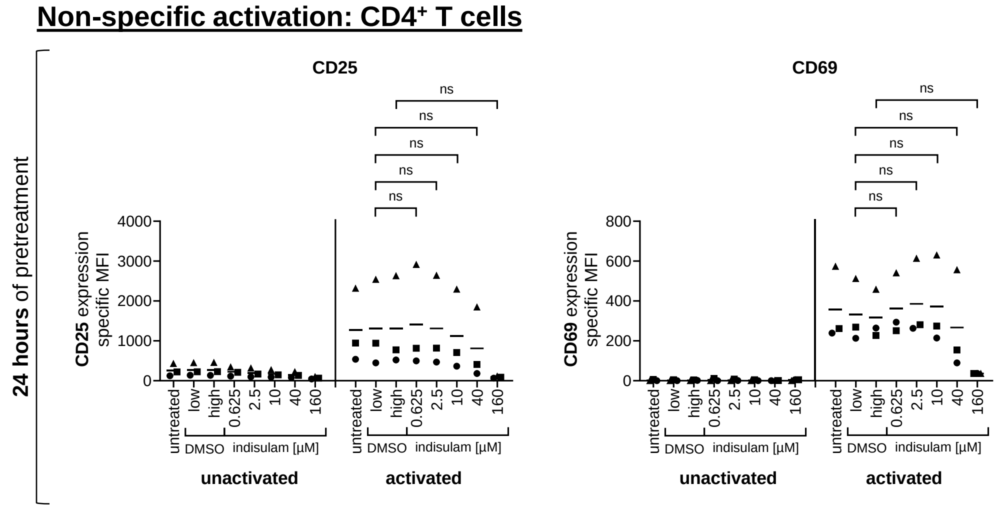

(a)

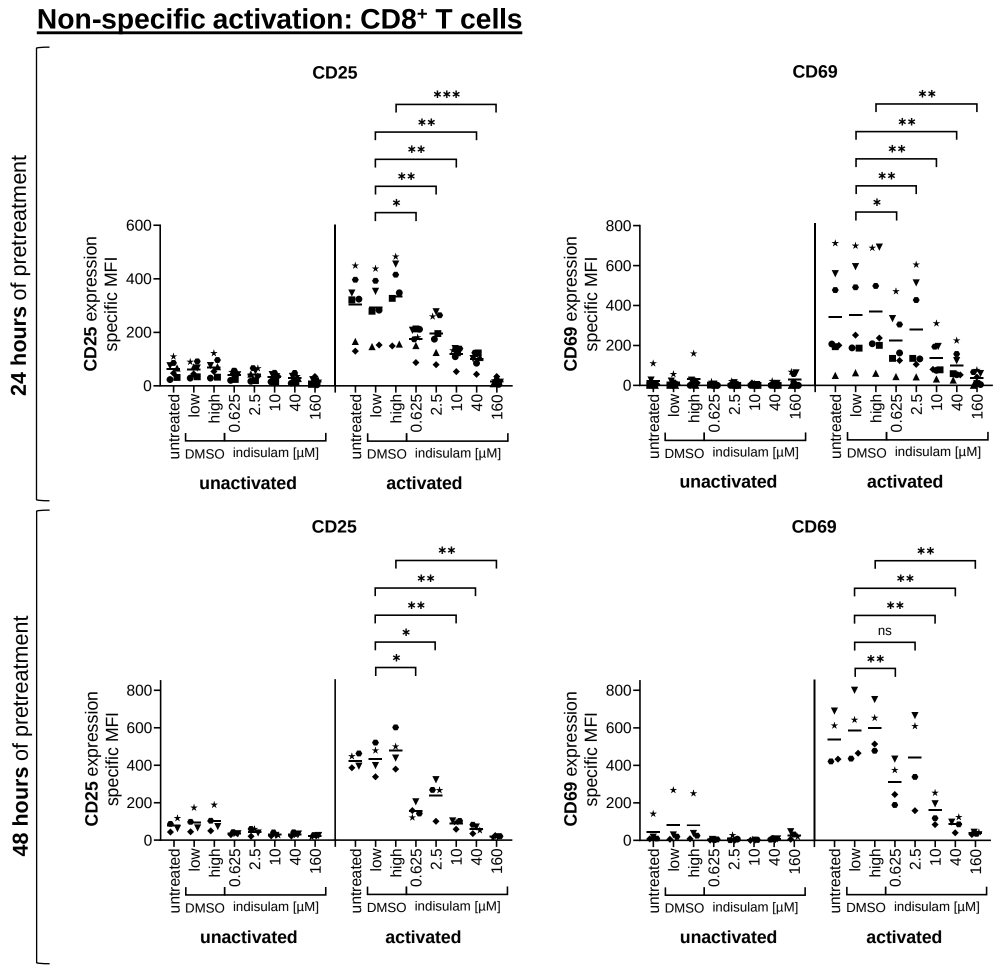

(b)

Supplemental Figure S1. Indisulam influences the expression of the activation markers CD25 and CD69 on T cells following non-specific activation (MFI). CD4+ (**a**) and CD8+ (**b**) T cells were either treated with the splicing inhibitor indisulam at the indicated concentrations, treated with the DMSO solvent controls (DMSO low equivalent to the quantity of DMSO contained in 40 µM indisulam, DMSO high equivalent to the quantity of DMSO contained in 160 µM indisulam), or left untreated. After treatment of approximately 24 h or 48 h, T cells were activated in the presence or absence of DMSO or indisulam. The non-specific T-cell activation was performed by CD3-crosslinking. After 20-24 hours of activation, T cells were harvested and living T cells were analyzed for the surface expression of the activation markers CD25 and CD69 using flow cytometry. The absolute expression of surface markers CD25 and CD69 in response to T-cell activation after treatment with DMSO or indisulam at different concentrations of a wide range (as indicated) is depicted. The expression of activation markers on T cells is displayed as specific MFI (i.e., MFI after subtracting background MFI of the respective unstained sample). Mean values (horizontal bars) are shown from three (CD4+, 24 h), seven (CD8+, 24 h), or four (CD8+, 48 h) different donors (represented as different symbols). *p*-values were calculated with paired Student's *t*-test using the values for absolute expression compared to the respective DMSO solvent control. The conditions up to 40 µM indisulam were tested against DMSO low, the condition with 160 µM indisulam was tested against DMSO high. * = significant (* $p \leq 0.05$, ** $p \leq 0.01$, *** $p \leq 0.001$, **** $p \leq 0.0001$), ns = not significant ($p > 0.05$).

Non-specific activation: CD4⁺ T cells

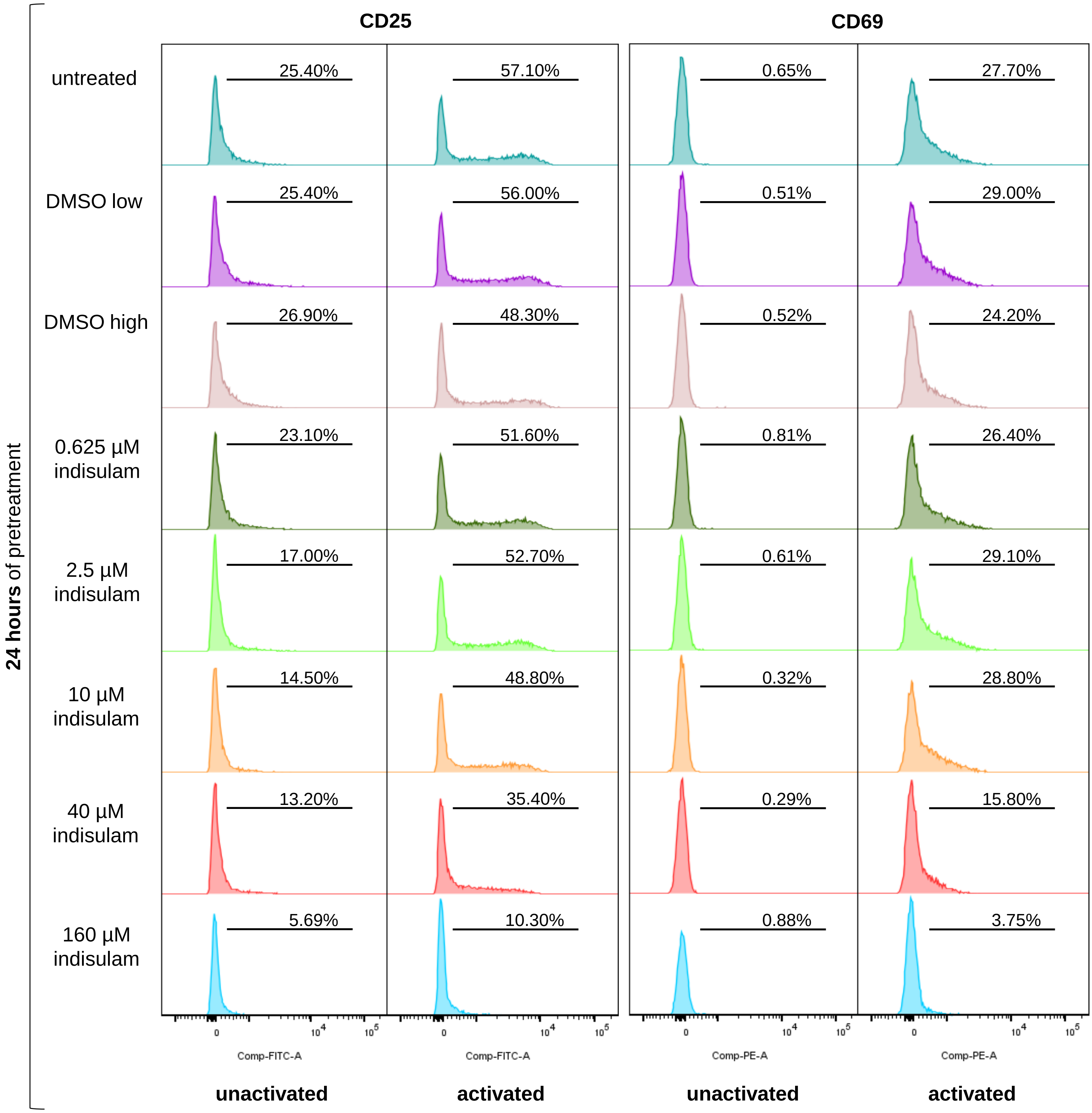

Supplemental Figure S2. Indisulam influences the expression of the activation markers CD25 and CD69 on CD4⁺ T cells following non-specific activation (24 h; % positive cells). CD4⁺ T cells were either treated with the splicing inhibitor indisulam at the indicated concentrations, treated with the DMSO solvent controls (DMSO low equivalent to the quantity of DMSO contained in 40 µM indisulam, DMSO high equivalent to the quantity of DMSO contained in 160 µM indisulam), or left untreated. After treatment of approximately 24 h, T cells were activated in the presence or absence of DMSO or indisulam. The non-specific T-cell activation was performed by CD3-crosslinking. After 20-24 hours of activation, T cells were harvested and living T cells were analyzed for the surface expression of the activation markers CD25 and CD69 using flow cytometry. Exemplary illustration of the detected fluorescence signals with the percentages of the positive population from one representative donor is shown.

Non-specific activation: CD8+ T cells

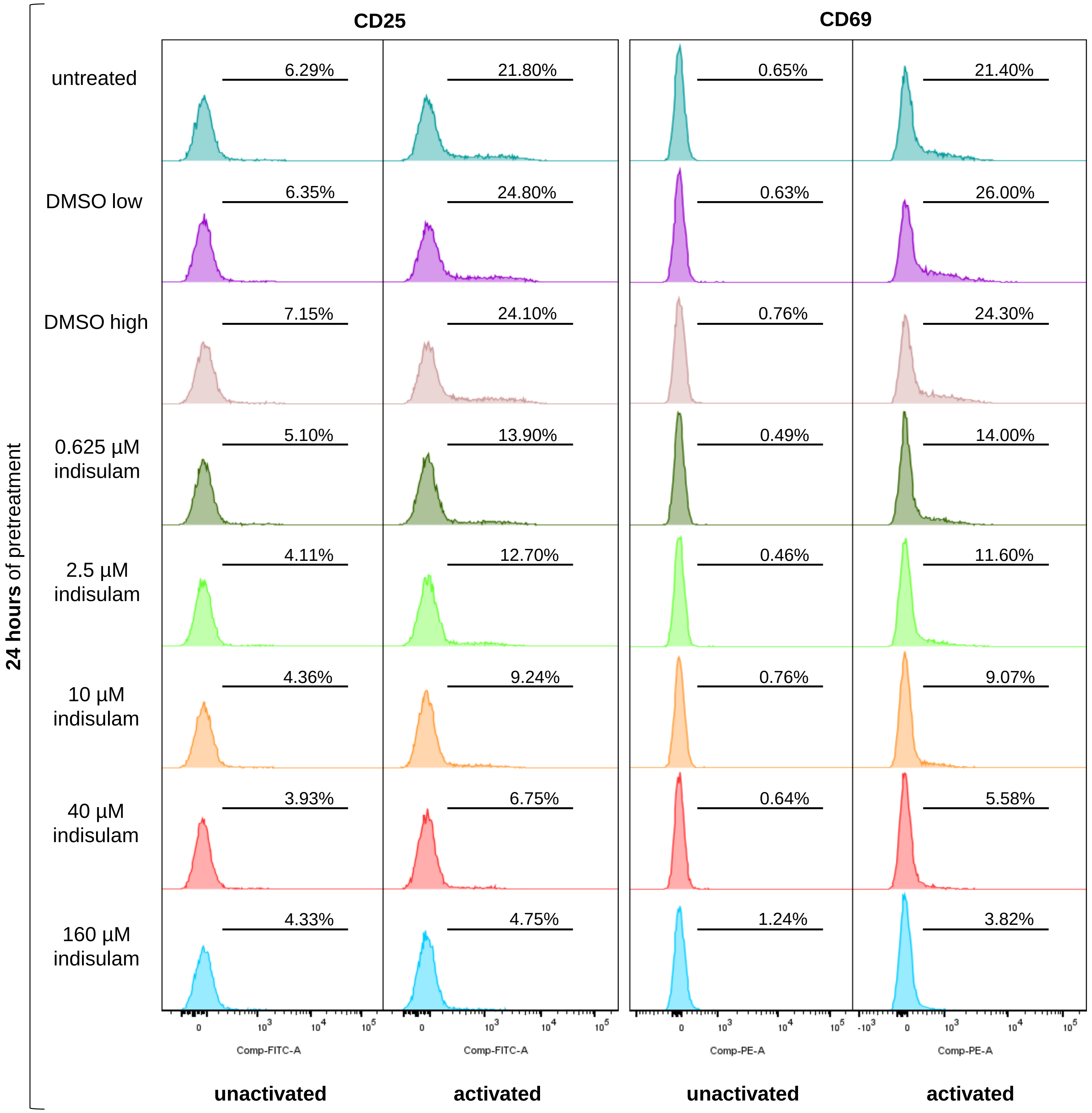

Supplemental Figure S3. Indisulam influences the expression of the activation markers CD25 and CD69 on CD8+ T cells following non-specific activation (24 h; % positive cells). CD8+ T cells were either treated with the splicing inhibitor indisulam at the indicated concentrations, treated with the DMSO solvent controls (DMSO low equivalent to the quantity of DMSO contained in 40 µM indisulam, DMSO high equivalent to the quantity of DMSO contained in 160 µM indisulam), or left untreated. After treatment of approximately 24 h, T cells were activated in the presence or absence of DMSO or indisulam. The non-specific T-cell activation was performed by CD3-crosslinking. After 20-24 hours of activation, T cells were harvested and living T cells were analyzed for the surface expression of the activation markers CD25 and CD69 using flow cytometry. Exemplary illustration of the detected fluorescence signals with the percentages of the positive population from one representative donor is shown.

Non-specific activation: CD8+ T cells

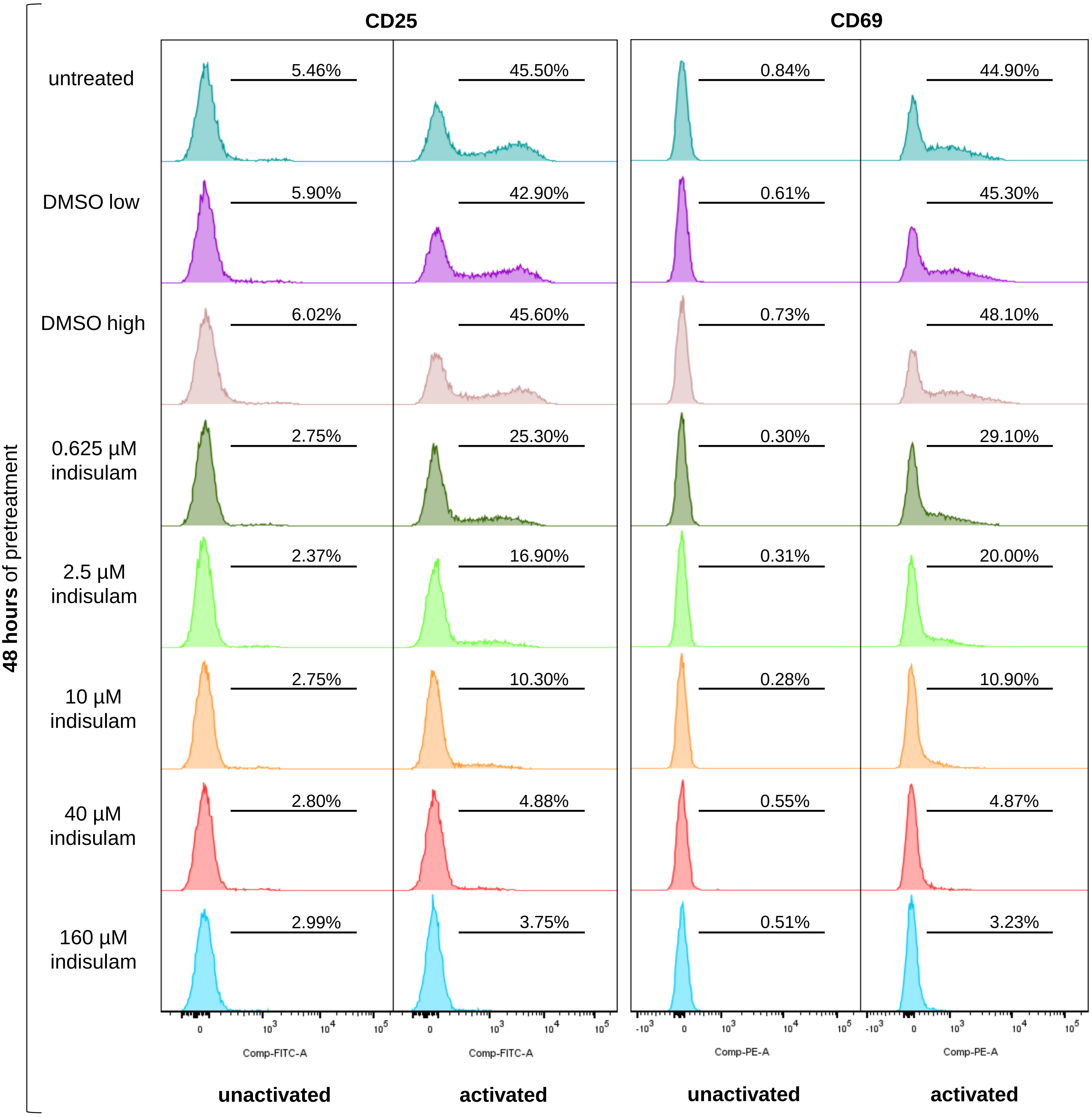

Supplemental Figure S4. Indisulam influences the expression of the activation markers CD25 and CD69 on CD8+ T cells following non-specific activation (48 h; % positive cells). CD8+ T cells were either treated with the splicing inhibitor indisulam at the indicated concentrations, treated with the DMSO solvent controls (DMSO low equivalent to the quantity of DMSO contained in 40 µM indisulam, DMSO high equivalent to the quantity of DMSO contained in 160 µM indisulam), or left untreated. After treatment of approximately 48 h, T cells were activated in the presence or absence of DMSO or indisulam. The non-specific T-cell activation was performed by CD3-crosslinking. After 20-24 hours of activation, T cells were harvested and living T cells were analyzed for the surface expression of the activation markers CD25 and CD69 using flow cytometry. Exemplary illustration of the detected fluorescence signals with the percentages of the positive population from one representative donor is shown.

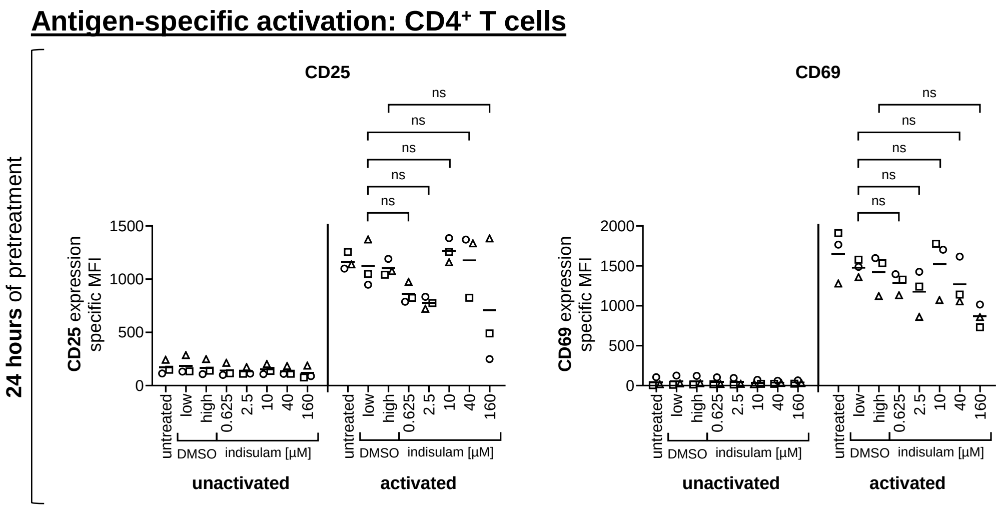

(**a**)

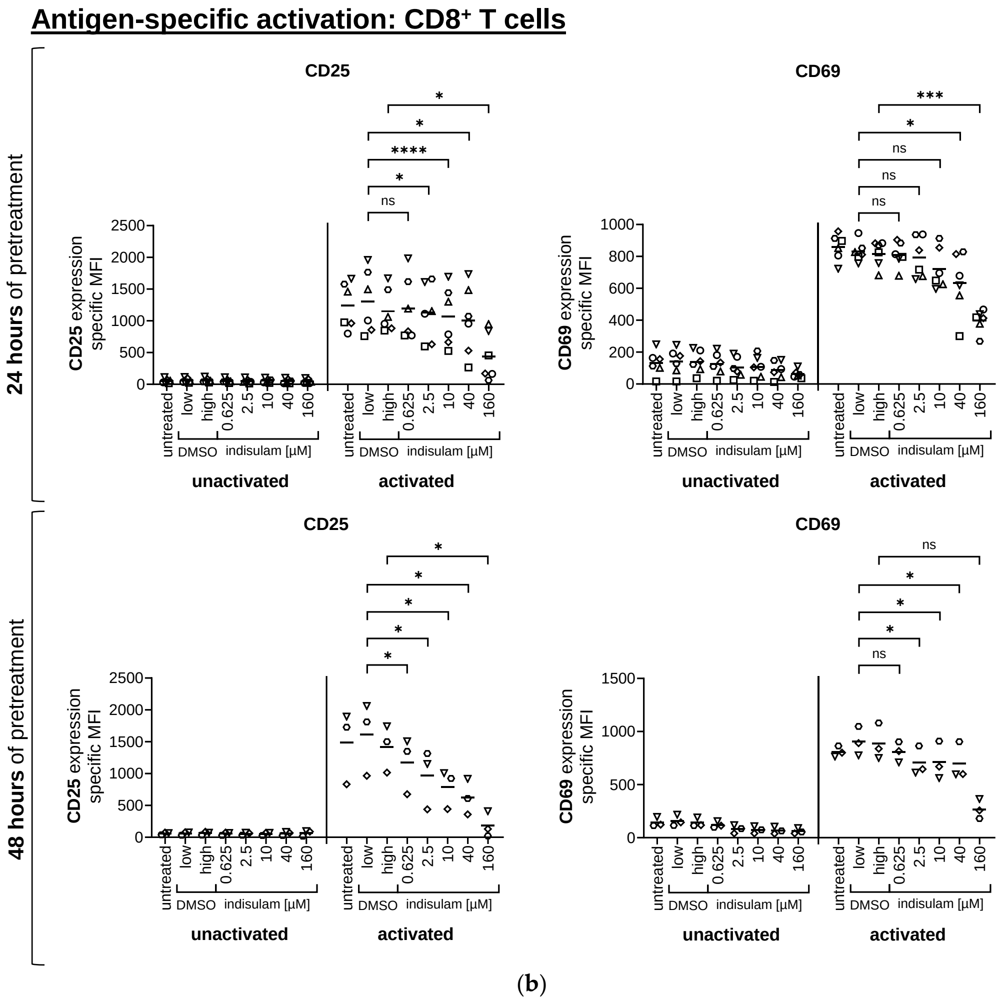

(**b**)

Supplemental Figure S5. Indisulam influences the expression of the activation markers CD25 and CD69 on T cells following antigen-specific activation (MFI). CD4+ (**a**) and CD8+ (**b**) T cells were either treated with the splicing inhibitor indisulam at the indicated concentrations, treated with the DMSO solvent controls (DMSO low equivalent to the quantity of DMSO contained in 40 µM indisulam, DMSO high equivalent to the quantity of DMSO contained in 160 µM indisulam), or left untreated. After treatment of approximately 24 h or 48 h, T cells were activated in the presence or absence of DMSO or indisulam. The antigen-specific T-cell activation was performed with T cells transfected with a gp100-specific TCR by co-incubating them with gp100-loaded target cells. After 20-24 hours of activation, T cells were harvested and living T cells were analyzed for the surface expression of the activation markers CD25 and CD69 using flow cytometry. The absolute expression of surface markers CD25 and CD69 in response to T-cell activation after treatment with DMSO or indisulam at different concentrations of a wide range (as indicated) is depicted. The expression of activation markers on T cells is displayed as specific MFI (i.e., MFI after subtracting background MFI of the respective unstained sample). Mean values (horizontal bars) are shown from three (CD4+, 24 h), six (CD8+, 24 h), or three (CD8+, 48 h) different donors (represented as different symbols). *p*-values were calculated with paired Student's *t*-test using the values for absolute expression compared to the respective DMSO solvent control. The conditions up to 40 µM indisulam were tested against DMSO low, the condition with 160 µM indisulam was tested against DMSO high. * = significant (* $p \le 0.05$, ** $p \le 0.01$, *** $p \le 0.001$, **** $p \le 0.0001$), ns = not significant ($p > 0.05$).

Antigen-specific activation: CD4+ T cells

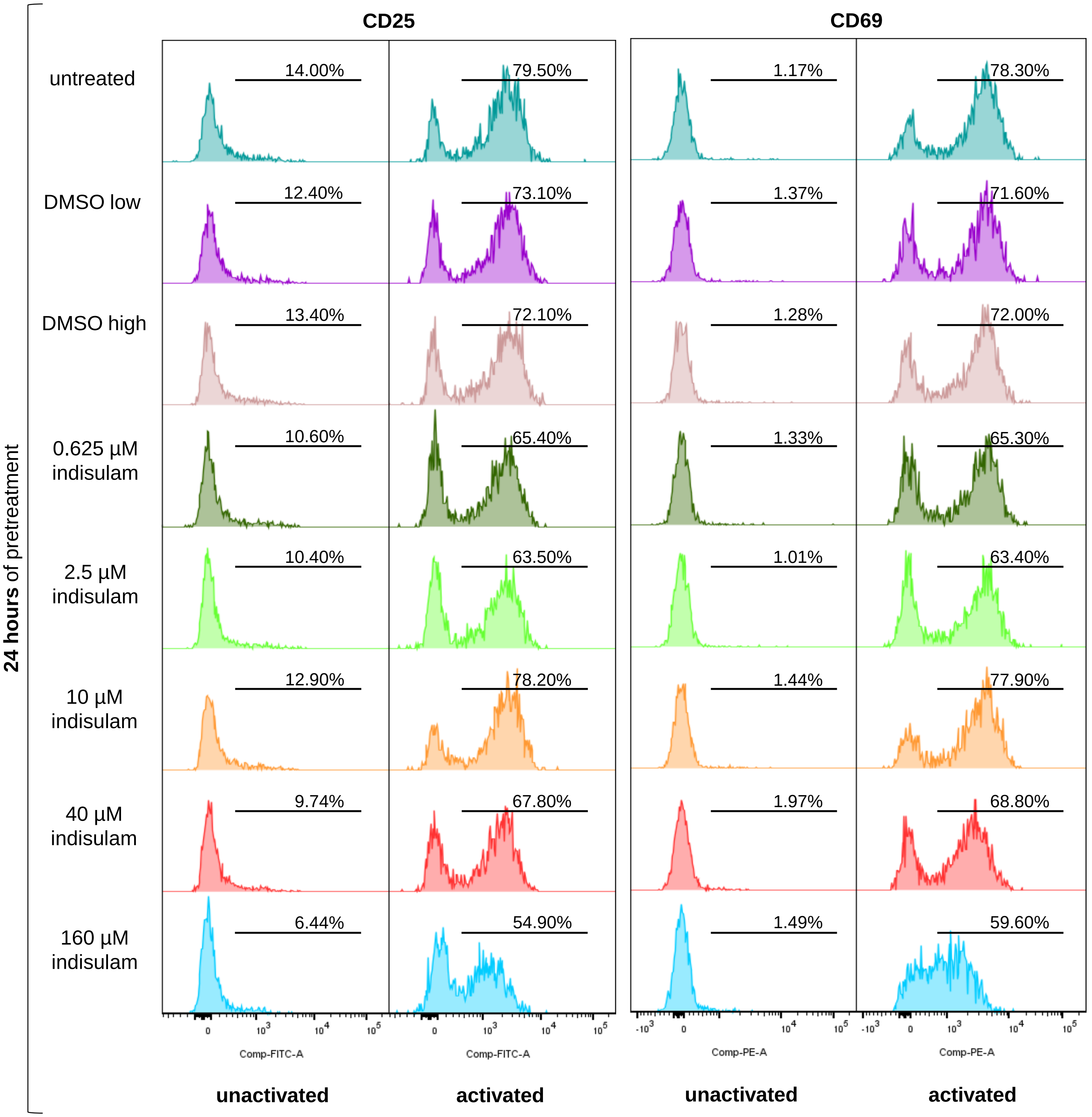

Supplemental Figure S6. Indisulam influences the expression of the activation markers CD25 and CD69 on CD4+ T cells following antigen-specific activation (24 h; % positive cells). CD4+ T cells were either treated with the splicing inhibitor indisulam at the indicated concentrations, treated with the DMSO solvent controls (DMSO low equivalent to the quantity of DMSO contained in 40 µM indisulam, DMSO high equivalent to the quantity of DMSO contained in 160 µM indisulam), or left untreated. After treatment of approximately 24 h, T cells were activated in the presence or absence of DMSO or indisulam. The antigen-specific T-cell activation was performed with T cells transfected with a gp100-specific TCR by co-incubating them with gp100-loaded target cells. After 20-24 hours of activation, T cells were harvested and living T cells were analyzed for the surface expression of the activation markers CD25 and CD69 using flow cytometry. Exemplary illustration of the detected fluorescence signals with the percentages of the positive population from one representative donor is shown.

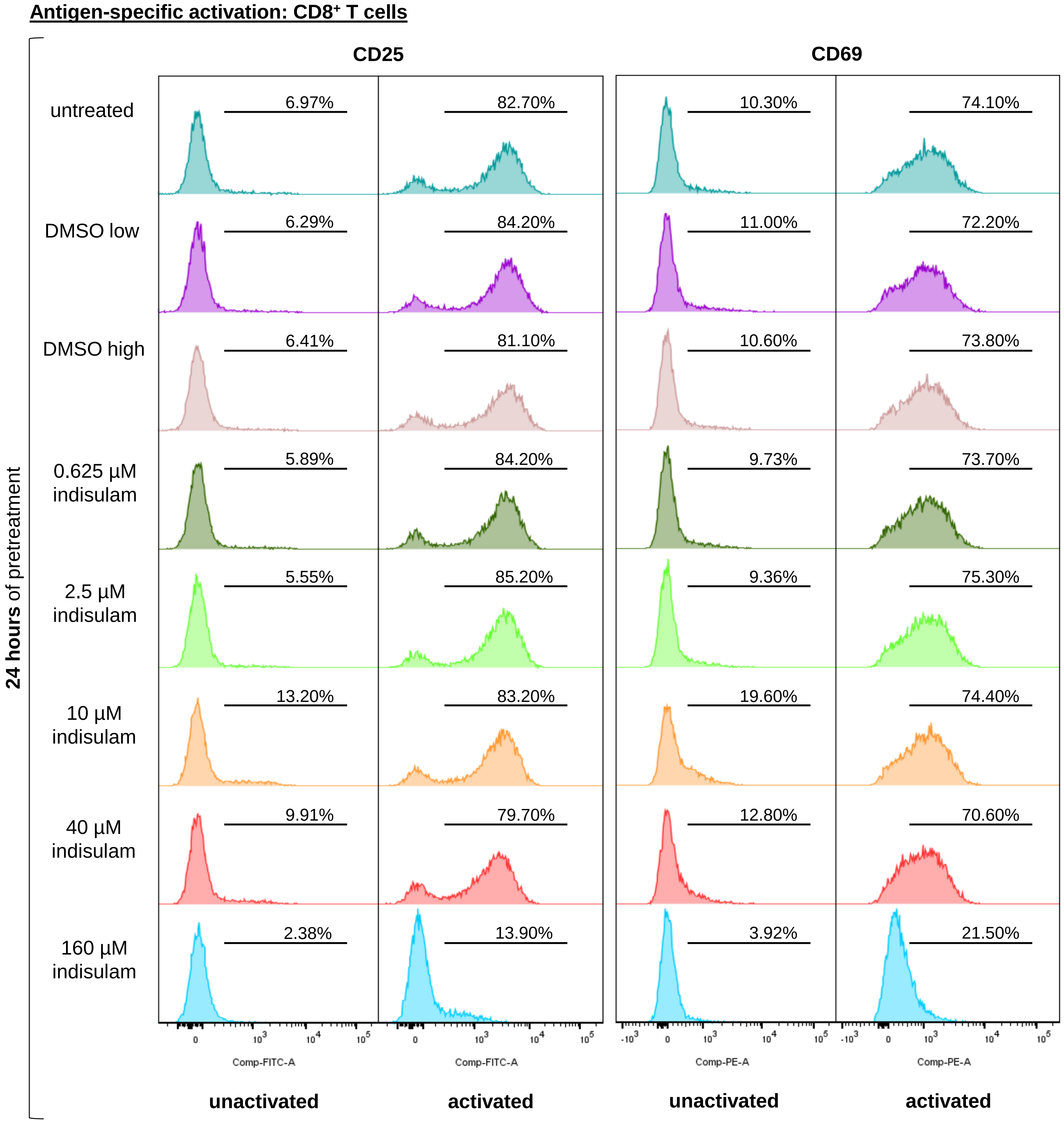

Supplemental Figure S7. Indisulam influences the expression of the activation markers CD25 and CD69 on CD8+ T cells following antigen-specific activation (24 h; % positive cells). CD8+ T cells were either treated with the splicing inhibitor indisulam at the indicated concentrations, treated with the DMSO solvent controls (DMSO low equivalent to the quantity of DMSO contained in 40 µM indisulam, DMSO high equivalent to the quantity of DMSO contained in 160 µM indisulam), or left untreated. After treatment of approximately 24 h, T cells were activated in the presence or absence of DMSO or indisulam. The antigen-specific T-cell activation was performed with T cells transfected with a gp100-specific TCR by co-incubating them with gp100-loaded target cells. After 20-24 hours of activation, T cells were harvested and living T cells were analyzed for the surface expression of the activation markers CD25 and CD69 using flow cytometry. Exemplary illustration of the detected fluorescence signals with the percentages of the positive population from one representative donor is shown.

Antigen-specific activation: CD8$^+$ T cells

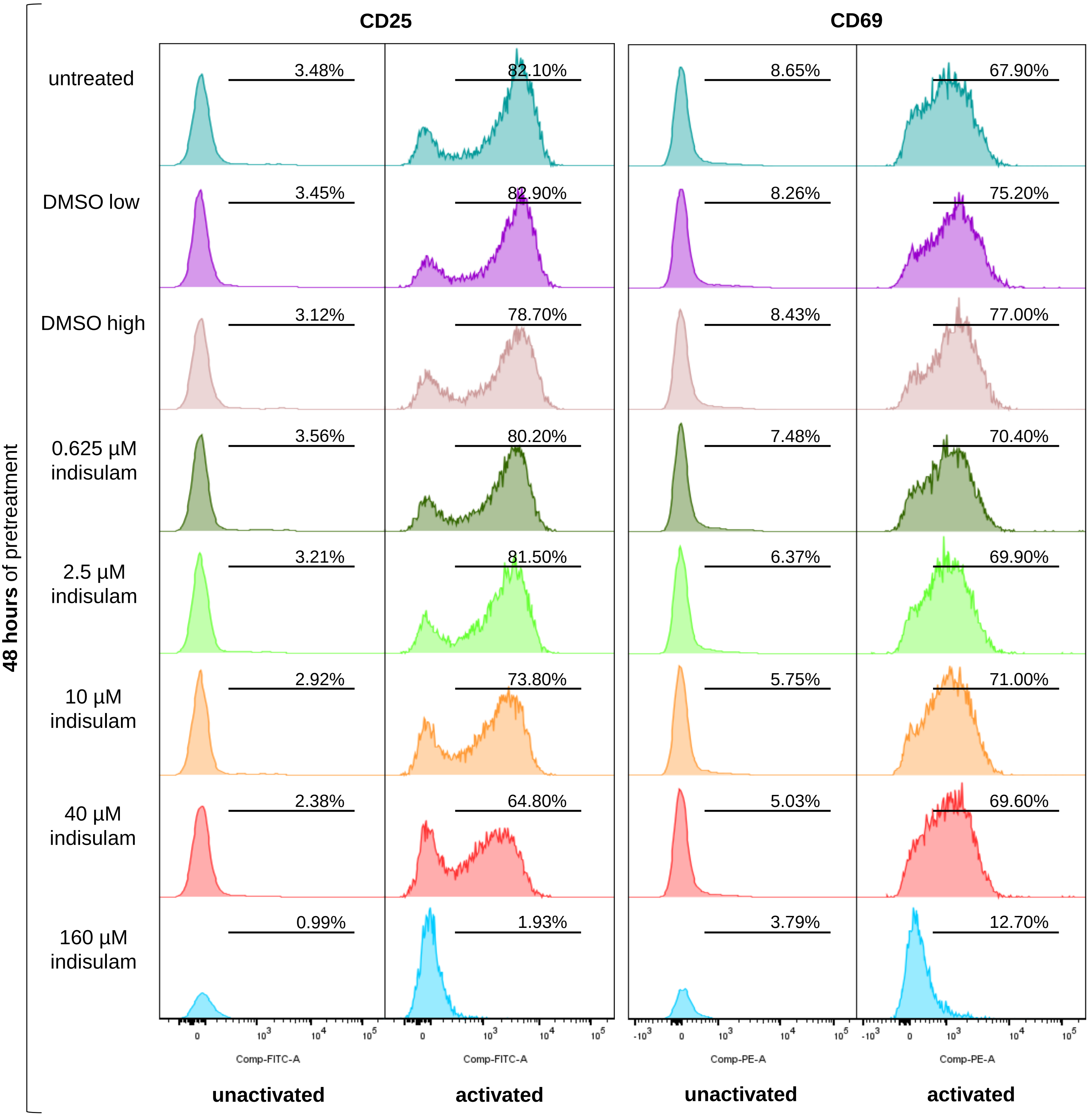

Supplemental Figure S8. Indisulam influences the expression of the activation markers CD25 and CD69 on CD8$^+$ T cells following antigen-specific activation (48 h; % positive cells). CD8$^+$ T cells were either treated with the splicing inhibitor indisulam at the indicated concentrations, treated with the DMSO solvent controls (DMSO low equivalent to the quantity of DMSO contained in 40 µM indisulam, DMSO high equivalent to the quantity of DMSO contained in 160 µM indisulam), or left untreated. After treatment of approximately 48 h, T cells were activated in the presence or absence of DMSO or indisulam. The antigen-specific T-cell activation was performed with T cells transfected with a gp100-specific TCR by co-incubating them with gp100-loaded target cells. After 20-24 hours of activation, T cells were harvested and living T cells were analyzed for the surface expression of the activation markers CD25 and CD69 using flow cytometry. Exemplary illustration of the detected fluorescence signals with the percentages of the positive population from one representative donor is shown.

Non-specific activation: CD4+ T cells

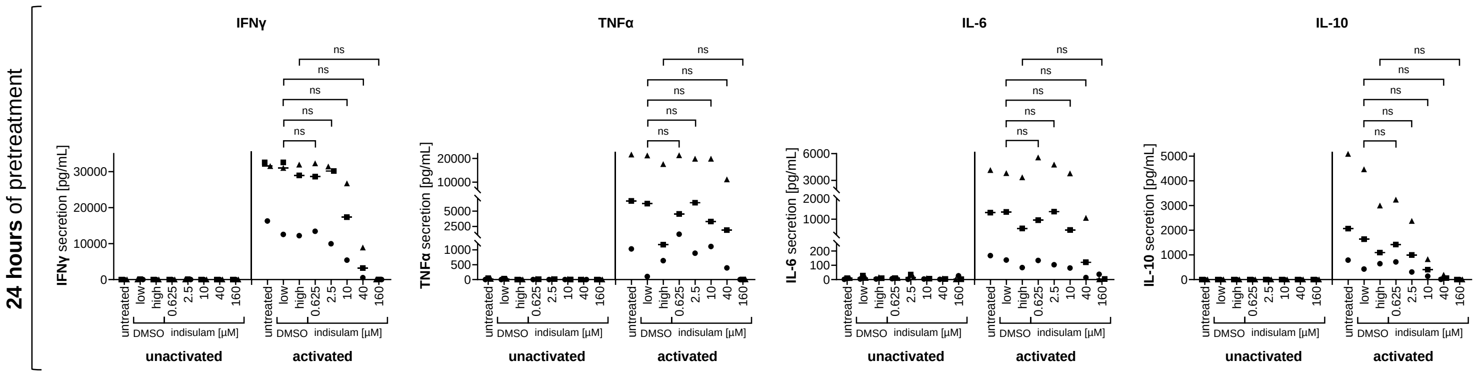

(**a**)

Non-specific activation: CD8+ T cells

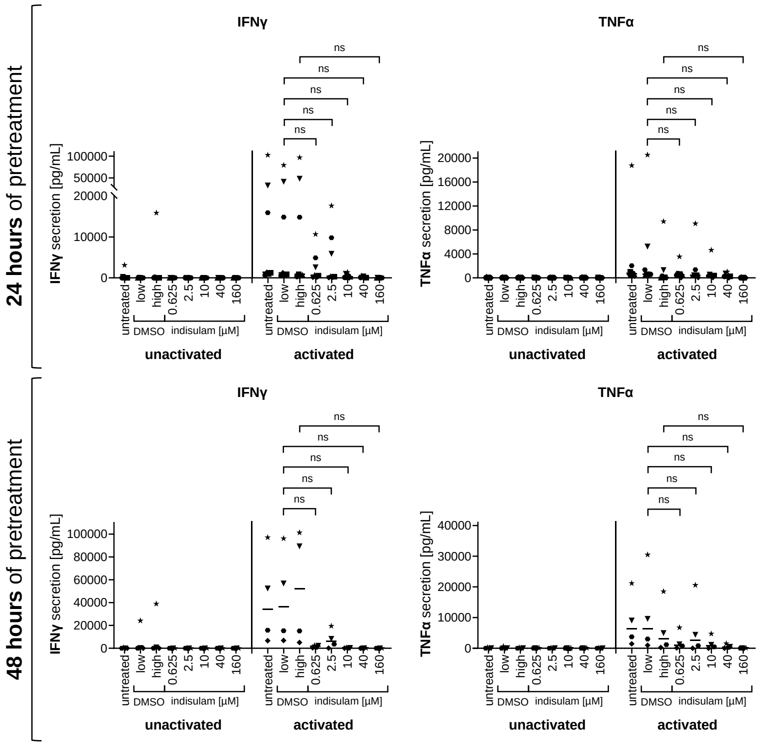

(**b**)

Supplemental Figure S9. Indisulam affects the cytokine secretion by activated T cells in a dose-dependent and time-dependent manner (non-specific activation). CD4+ (**a**) and CD8+ (**b**) T cells were either treated with the splicing inhibitor indisulam at the indicated concentrations, treated with the DMSO solvent controls (DMSO low equivalent to the quantity of DMSO contained in 40 µM indisulam, DMSO high equivalent to the quantity of DMSO contained in 160 µM indisulam) or were left untreated (i.e. no indisulam, no DMSO). After treatment of approximately 24 h or 48 h, T cells were activated in the presence or absence of DMSO or indisulam. The non-specific T-cell activation was performed by CD3-crosslinking. After 20-24 hours of T-cell activation, the supernatants were collected and analyzed for secreted cytokines using a cytometric bead array. The absolute cytokine concentration in response to T-cell activation after treatment with DMSO or indisulam at different concentrations of a wide range (as indicated) is depicted. Mean values (horizontal bars) are shown from three (CD4+, 24 h), seven (CD8+, 24 h), or four (CD8+, 48 h) different donors (represented as different symbols). *p*-values were calculated with paired Student's *t*-test using the values for cytokine concentration compared to the respective DMSO solvent control. The conditions up to 40 µM indisulam were tested against DMSO low, the condition with 160 µM indisulam was tested against DMSO high. * = significant (* $p \leq 0.05$, ** $p \leq 0.01$, *** $p \leq 0.001$, **** $p \leq 0.0001$), ns = not significant ($p > 0.05$).

Antigen-specific activation: CD4+ T cells

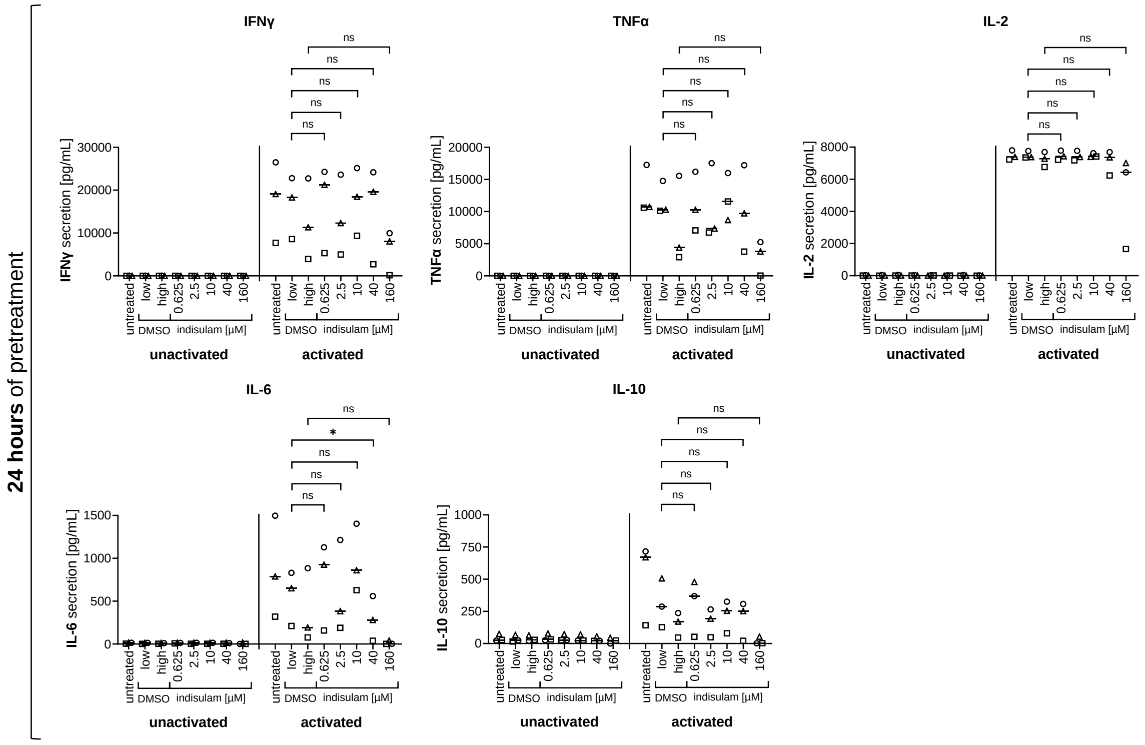

(**a**)

Antigen-specific activation: CD8+ T cells

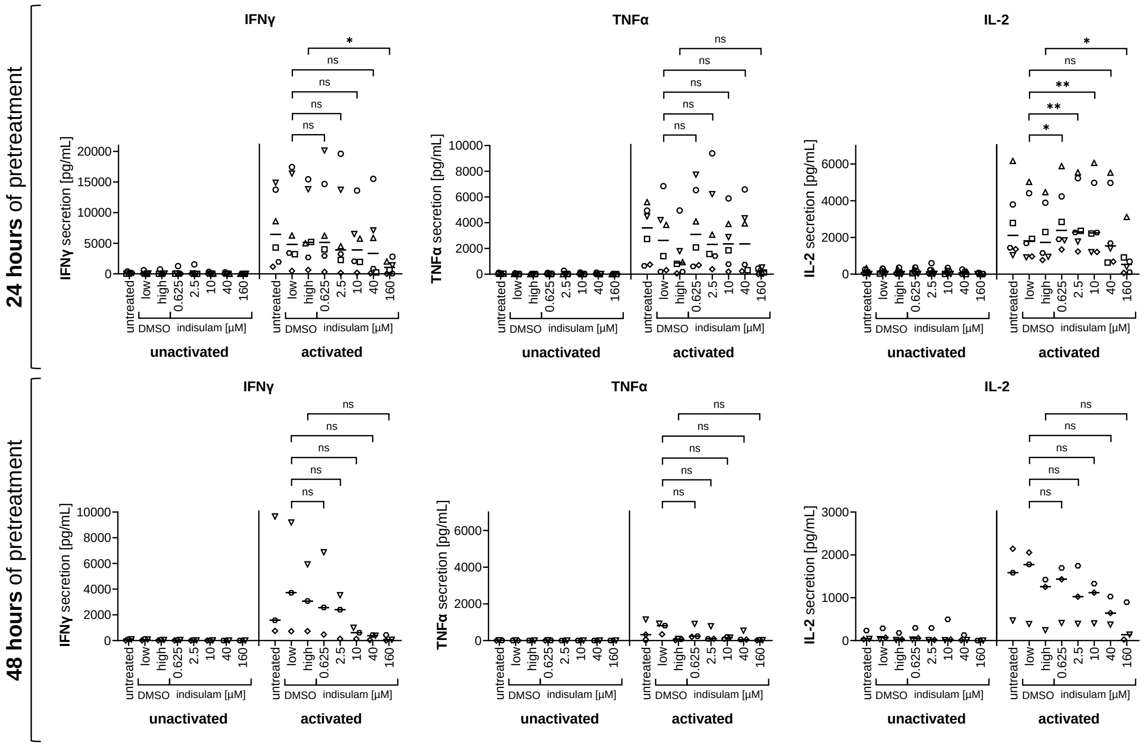

(**b**)

Supplemental Figure S10. Indisulam affects the cytokine secretion by activated T cells in a dose-dependent and time-dependent manner (antigen-specific activation). CD4+ (**a**) and CD8+ (**b**) T cells were either treated with the splicing inhibitor indisulam at the indicated concentrations, treated with the DMSO solvent controls (DMSO low equivalent to the quantity of DMSO contained in 40 µM indisulam, DMSO high equivalent to the quantity of DMSO contained in 160 µM indisulam) or were left untreated (i.e. no indisulam, no DMSO). After treatment of approximately 24 h or 48 h, T cells were activated in the presence or absence of DMSO or indisulam. The antigen-specific T-cell activation was performed using T cells transfected with a gp100-specific TCR and co-incubated with gp100-loaded target cells. After 20 24 hours of T-cell activation, the supernatants were collected and analyzed for secreted cytokines using a cytometric bead array. The absolute cytokine concentration in response to T-cell activation after treatment with DMSO or indisulam at different concentrations of a wide range (as indicated) is depicted. Mean values (horizontal bars) are shown from three (CD4+, 24 h), six (CD8+, 24 h), or three (CD8+, 48 h) different donors (represented as different symbols). *p*-values were calculated with paired Student's *t*-test using the values for cytokine concentration compared to the respective DMSO solvent control. The conditions up to 40 µM indisulam were tested against DMSO low, the condition with 160 µM indisulam was tested against DMSO high. * = significant (* $p \leq 0.05$, ** $p \leq 0.01$, *** $p \leq 0.001$, **** $p \leq 0.0001$), ns = not significant ($p > 0.05$).

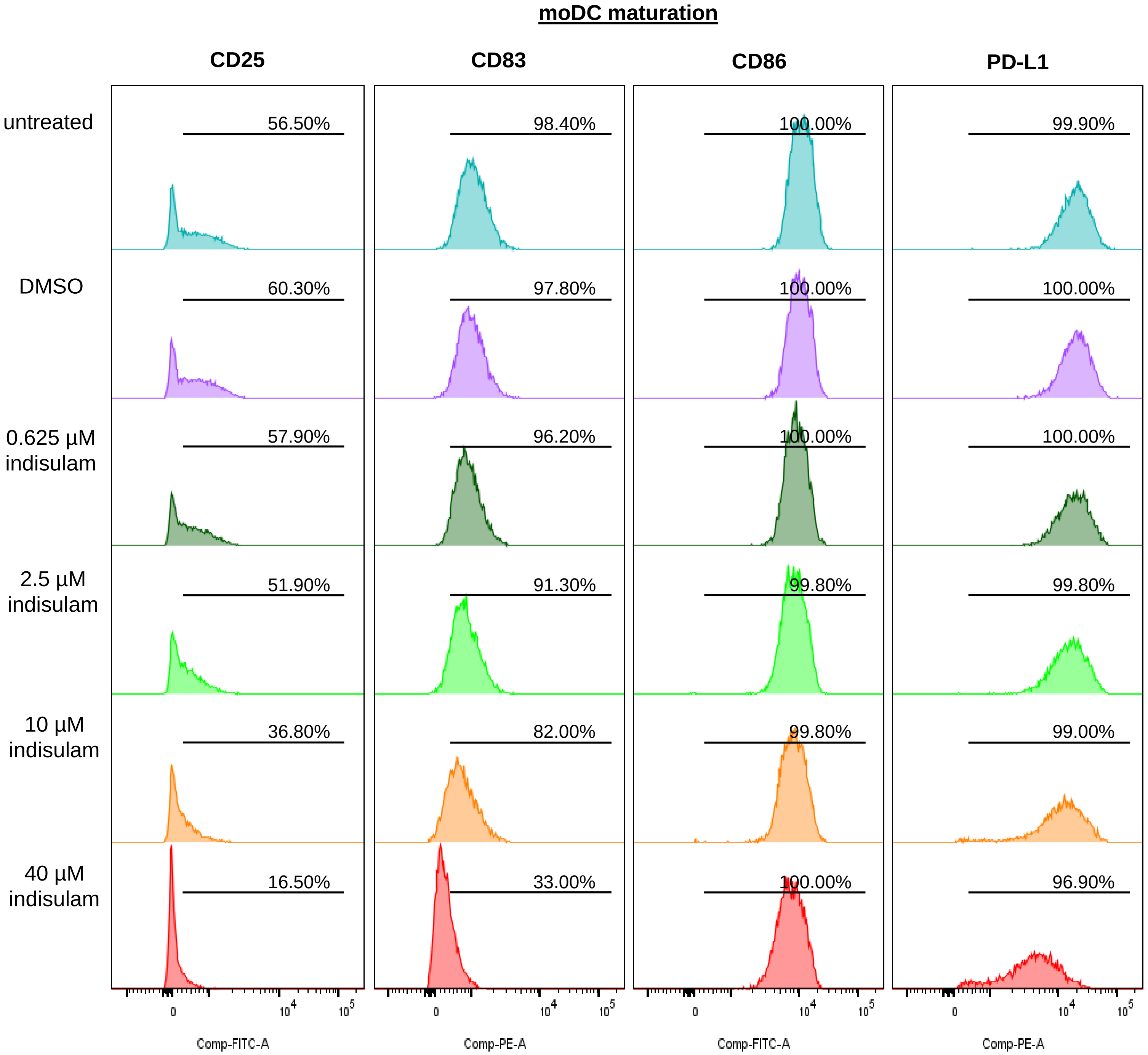

Supplemental Figure S11. The maturation-mediated upregulation of certain surface markers on moDCs is inhibited by high indisulam concentrations, but only moderately by low indisulam concentrations. Immature moDCs were treated with the splicing inhibitor indisulam at the indicated concentrations or treated with the DMSO solvent control (equivalent to the quantity of DMSO contained in the condition with 40 µM indisulam). After 24 hours of treatment, moDCs were matured in the presence or absence of indisulam with a standard cytokine cocktail containing IL-1β, PGE_2, IL-6, and TNFα. After 24 hours of maturation, moDCs were harvested and analyzed for surface expression of the maturation markers CD25, CD83, CD86, and PD-L1 using flow cytometry. Exemplary illustration of the detected fluorescence signals with the percentages of the positive population from one representative donor is shown.
